# Intestinal Inflammation and Parkinson’s Disease

**DOI:** 10.14336/AD.2021.0418

**Published:** 2021-12-01

**Authors:** Yu Li, Yuanyuan Chen, Lili Jiang, Jingyu Zhang, Xuhui Tong, Dapeng Chen, Weidong Le

**Affiliations:** ^1^Comparative Medicine Department of Researching and Teaching, Dalian Medical University, Dalian, China.; ^2^Liaoning Provincial Key Laboratory for Research on the Pathogenic Mechanisms of Neurological Diseases, the First Affiliated Hospital, Dalian Medical University, Dalian, Liaoning, China; ^3^Institute of Neurology, Sichuan Academy of Medical Science-Sichuan Provincial Hospital, Chengdu, Sichuan, China

**Keywords:** Inflammation, inflammatory bowel disease, microbiota, Parkinson’s disease

## Abstract

Parkinson’s disease (PD) is the second most common neurodegenerative disease which significantly influences the life quality of patients. The protein α-synuclein plays an important driving role in PD occurrence and development. Braak’s hypothesis suggests that α-synuclein is produced in intestine, and then spreads into the central nervous system through the vagus nerve. The abnormal expression of α-synuclein has been found in inflammatory bowel disease (IBD). Intestinal inflammation and intestinal dysbiosis have been involved in the occurrence and development of PD. The present review aimed to summarize recent advancements in studies focusing on intestinal inflammation and PD, especially the mechanisms through which link intestinal inflammation and PD. The intestinal dysfunctions such as constipation have been introduced as non-motor manifestations of PD. The possible linkages between IBD and PD, including genetic overlaps, inflammatory responses, intestinal permeability, and intestinal dysbiosis, are mainly discussed. Although it is not confirmed whether PD starts from intestine, intestinal dysfunction may affect intestinal microenvironment to influence central nervous system, including the α-synuclein pathologies and systematic inflammation. It is expected to develop some new strategies in the diagnosis and treatment of PD from the aspect of intestine. It may also become an exciting direction to find better ways to regulate the composition of gut microorganism to treat PD.

Parkinson’s disease (PD) is the second most common neurodegenerative disease pathologically characterized by the loss of dopaminergic neurons in the substantia nigra compacta. PD is clinically manifested by both motor and non-motor symptoms. The classic motor symptoms, including shaking, rigidity, slowness of movement, postural instability and difficulty with walking and gait, are the basis of current PD diagnosis [1].

As one of non-motor manifestations in PD, intestinal dysfunction has the prevalence as high as 77%-81% [2], including constipation, small intestinal bacterial overgrowth (SIBO), irritable bowel syndrome (IBS), *Helicobacter pylori* (*H. pylori*) infection, diarrhea and inflammatory bowel disease (IBD) [3]. Constipation is the most common and the most distressing intestinal symptom in PD compared with other intestinal dysfunctions [4].

The protein α-synuclein is the major constituent of Lewy bodies which is the primary pathological hallmark of PD. The appearance of misfolded α-synuclein occurs early in the gut mucosa before the formation of Lewy bodies in the brain [5], but the trigger of α-synuclein is unknown. According to Break’s hypothesis, the α-synuclein pathology spreads from the enteric plexuses to the central nervous system (CNS) through the vagus nerve, thereafter leads to PD [6]. Post-mortem studies have shown the presence of aggregated α-synuclein in the enteric nervous system (ENS) [7, 8], especially in the appendix [9]. Patients who had appendectomies showed a delayed age of PD onset [10], suggesting that the normal human appendix contains pathogenic forms of α-synuclein. The trigger of intestinal α-synuclein in intestinal tract under pathological state was rarely studied. It has been shown that α-synuclein also aggregates in the ENS of IBD patients and almost none in healthy individuals [11]. Recently, increased expression of α-synuclein is also found in in both colonic submucosal and myenteric plexus of CD patients [12].

Studies in Danish and Taiwanese have reported an increased prevalence and relatively higher risk of PD amongst patients with IBD [13, 14]. Another two studies have also shown that IBD is associated with an increased risk of PD [15, 16], whereas a lower risk of PD has been observed in IBD patients who were under anti-inflammatory treatment [16, 17]. In addition, the risk of developing PD in IBD patients might be age-dependent. A recent search reported that those with later age seem to be associated with increased risk of PD [18]. However, data from several studies indicate that PD may be inversely associated with IBD overall [19, 20], and there are also studies suggest that the communication is bidirectional [21].

The intestinal inflammatory processes have been found to occur in PD patients, which have naturally predicted a possible association between intestinal inflammation and PD [22]. Hence, in this review, we aimed to summarize the research progress in correlations between IBD and PD (Table 1).

**Table 1:** Possible linkages between IBD and PD.

	Possible linkages between IBD and PD
Intestinal dysfunctions in PD	Constipation, *H. pylori* infection, small intestinal bacterial over growth, irritable bowel syndrome; diarrhea, intestinal inflammation, intestinal endotoxemia
Genetic overlap (Mainly the genes whose functions in IBD/PD have been studied)	*LRRK2, CARD15/NOD2, TLR, Nurr1, NLRP3, GBA, GPR65, SLC39A8*
Inflammation process	Similar pathological process Intestinal injury may induce systemic inflammation and subsequent neurodegeneration
Increased intestinal permeability	Downregulated tight junction expression. Elevated urinary sucralose level with normal mannitol and lactulose levels. Increased fecal markers.
Intestinal dysbiosis	Reduced bacterial diversity. Increased bacterial: *Lactobacillus*, Enterobacteriaceae and *Proteus*. Decreased bacterial: *Coprococcus*, *Faecalibacterium Roseburia*, Prevotellaceae, *Faecalibacterium prausnitzii*, Lachnospiraceae and *Ruminococcus.*

IBD, inflammatory bowel disease; PD, Parkinson’s disease; *H. pylori, Helicobacter pylori*; *LRRK2, Leucine-rich repeat kinase 2*; *TLR, Toll-like receptor*; *Nurr1, Nuclear receptor-related factor 1*; *NLRP3, NLR Family Pyrin Domain Containing 3*; *GBA, β-glucocerebrosidase*; *GPR65, G-protein coupled receptor 65*; *SLC39A8, Solute Carrier Family 39 Member 8*.

## Intestinal dysfunctions in PD

**Figure 1.**
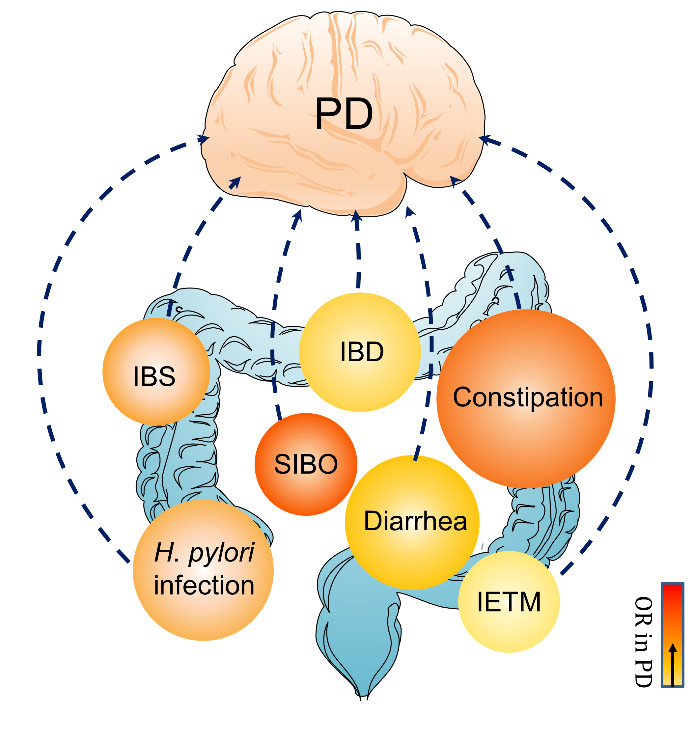
Intestinal dysfunctions in PD. Intestinal dysfunctions occurring in PD are marked in bubbles. The color level of the bubbles represents the OR of intestinal dysfunctions in PD. IBD, inflammatory bowel disease; IBS, irritable bowel syndrome, IETM: intestinal endotoxemia, *H. pylori*: *Helicobacter pylori*, OR: odd ratio.

Intestinal dysfunctions that observed in PD patients (Fig.1) include, but not limited to, constipation, SIBO, IBS, *H. pylori* infection, diarrhea, intestinal inflammation and IETM. And intestinal endotoxemia (IETM) is observed in animal models of PD. It still remains open whether these intestinal dysfunctions are actual risk factors or prodromal symptoms of PD.

### Constipation

Constipation is the most common and distressing intestinal symptom in PD which also occurs in IBD [4, 23]. Increased prevalence of constipation in patients with PD ranges from 24.6% to 63% [24].

Multiple factors have been involved in constipation in PD patients, including slow transit, anorectal dysfunction [25], and decreased vasoactive intestinal polypeptide expression in submucosal neurons [26]. It has been reported that constipation is due to a nondopaminergic pathology in PD [27], suggesting it as a non-motor sign but not a result of PD. Fecal microbiota transplantation (FMT) has been used for the treatment of chronic constipation with clinical success as the gut microbiota influences gut motility [28] and FMT also relieves constipation in PD [29, 30].

### *H. pylori* infection

*H. pylori* is a gram-negative bacterium that chronically colonizes the stomach and duodenal lining of more than 50% of the human population worldwide [31]. Altschuler [32] has hypothesized that *H. pylori* infection may be a cause of PD, while several studies have shown a higher prevalence of *H. pylori* infection in PD patients [33-35].

Patients with PD have an excessive number of peptic ulcers [36] and *H. pylori* mostly causes gastritis and peptic ulcer disease [37]. The mechanisms of the association between *H. pylori* and PD are not well understood, but are likely to be multifactorial. Several hypotheses of how this organism might contribute to PD development have been provided [38], including toxin(s) produced by the bacteria, disruption of the microbiota and local inflammation to neuroinflammation through the gut-brain axis. A review article has hypothesized that duodenal *H. pylori* infection might affect levodopa bioavailability by reducing levodopa absorption or inactivating the drug as the duodenum is the main site for levodopa absorption [34]. Likewise, *H. pylori* infection is associated with a poor response to levodopa and increased medication usage in PD patients [39, 40], while *H. pylori* eradication improves the levodopa and the motor severity of PD patients [41].

### SIBO

SIBO is a malabsorption syndrome characterized by a quantitative increase in the amount of small intestinal bacteria. Common symptoms of SIBO include bloating, abdominal pain, flatulence, diarrhea, and constipation, and the symptoms of SIBO can mimic those of IBD [42]. Patients with PD exhibit a highly increased prevalence of SIBO [43-45].

The main mechanisms that restrict bacterial colonization in the upper gastrointestinal tract are the gastric acid barrier, immunity, and intestinal motility. When one or more of these mechanisms fail, bacterial overgrowth develops [46]. The small intestine is particularly susceptible to the migration of bacteria and unlike the colon, it is not protected by a thick mucus barrier. Thus, bacterial overgrowth increases intestinal permeability, leading to excessive stimulation of inflammatory responses [47]. SIBO eradication also has a meaningful clinical effect on PD patients even in the setting of a concomitant *H. pylori* infection [45].

### IBS

IBS is a common, lifelong gastrointestinal motility disorder. Several epidemiological studies have considered IBS as a risk factor of PD. The overall incidence of PD in patients with IBS has been found to be 1.76-fold higher than that in non-IBS patients in Taiwan [48] and higher prevalence of IBS has also been observed in PD [49]. Recently, it has been supported by a retrospective registry-based cohort study identifying 28, 150 patients diagnosed with IBS during the years 1998-2014, showing that diagnosis of IBS is associated with a higher hazard of PD [50].

In patients with IBS, the intestinal mucosal cells and enteroendocrine cells secrete higher levels of proinflammatory cytokines and acute phase reaction proteins, thereby increasing the intestinal mucosal permeability [3]. The elevated level of bacteria triggers the production of proinflammatory cytokines by the enteric neurons, which are transmitted through the vagus nerve to the brain, where they cause neuroinflammation, which is one of the underlying mechanisms of PD [51].

### Diarrhea

The most common symptom of IBD is diarrhea, which is thought to result from changes in electrolyte transport associated with intestinal inflammation. In a meta-analysis on intestinal disorders in patients with PD, the OR of diarrhea was 1.27 [3], suggesting its potential role in the occurrence of PD.

In an investigation of intestinal dysfunctions in the model of rotenone-induced PD, these rats experienced more days with diarrhea [52]. Because diarrhea is a symptom of colitis, inflammation-induced colonic mucosal thickening in rotenone rats may cause diarrhea. Bile acid is another possible factor. Bile acids are important regulators of intestinal ?uid and electrolyte transport [53]. In conditions associated with bile acid malabsorption, their delivery to the colon at high concentrations induces ?uid and electrolyte secretion [54], thereby causing diarrhea. Of note, secondary bile acids are found to be increased in PD, suggesting the potential role of bile acid metabolism dysfunction in diarrhea [53].

### Intestinal inflammation

Several findings have provided evidence that intestinal inflammation occurs in PD. The most direct confirmation of intestinal inflammation in PD is the increase in mRNA transcripts encoding four proinflammatory cytokines as well as three glial markers in colonic biopsies of PD as compared to healthy controls [22]. High level of stool immune factors has further indicated that intestinal inflammation is present in patients with PD [55].

Several epidemiological studies have shown the prevalence of PD in patients with IBD [13-16]. Moreover, intestinal inflammation plays a substantial role in developing PD (reviewed in “Inflammation in both IBD and PD” below).

### IETM

Endotoxemia mainly results from large quantities of endotoxins produced by gram-negative bacteria in gut that are absorbed into portal veins because of the increased permeability of the intestinal wall and if the level of endotoxins exceeds the hepatic capacity for endotoxin scavenging due to decreased phagocytic ability of liver Kupffer cells (KCs), endotoxins may spill over into systemic circulation [56].

Lipopolysaccharide (LPS) is increased in both intestinal inflammation and neurodegenerative diseases and is used to induce neuroinflammation animal models [57, 58]. It has been reported that chronic stress-induced gut dysfunction exacerbates endotoxemia in a rotenone-induced mouse model of PD and enhances the phenotype and pathology of PD [59]. Because of the damaged gut permeability and decreased phagocytic ability of liver KCs, the increased level of LPS get to transfer from the gut to the plasma, resulting in systemic inflammation and secondary neuroinflammation.

### Genetic overlap between IBD and PD

In recent years, genetic analyses have been remarkably successful in identifying many common risk variants. The genetic overlaps between intestinal inflammation and PD may possibly imply potential pathophysiological roles in both IBD and PD. In 2017, genome-wide association studies (GWAS) and pathway analyses on patients with PD or seven autoimmune diseases, including CD and UC identified 17 novel pleiotropic gene loci [60]. The strongest genome-wide pleiotropic enrichment was found between PD and CD. Besides the human leucocyte antigen locus, six PD loci are reported associated with CD and three loci with UC. According to the National Genome Research Institute GWAS Catalog [61], six loci are strongly associated with PD and IBD, as evaluated by Ho-Su Lee et al [62]. However, the question is whether genetic polymorphisms play a role in linking both diseases and how they affect functionality. In this section, we summarized some typical genes mentioned above which protein encoded may functionally link IBD and PD. Besides those genes, we also summarized some potential genes including *Toll-like receptor (TLR), Nuclear receptor-related factor 1 (Nurr1), NLR Family Pyrin Domain Containing 3 (NLRP3) and Solute Carrier Family 39 Member 8 (SLC39A8)* as briefly shown in Table 2.

### *Leucine-rich repeat kinase 2* (*LRRK2*)

*LRRK2* has emerged as the most commonly associated gene with both familial and sporadic PD [63]. In GWAS, *LRRK2* has also been identified as a susceptibility gene for both CD and UC, especially CD [64]. LRRK2 is a large protein with several identifiable domains, including a Ras of complex proteins domain, a C-terminal of Roc domain, a kinase domain, and a leucine-rich repeat domain. LRRK2 is constitutively expressed in neurons and glial cells [65], and is high in immune cells, suggesting that its role in immune system [66].

LRRK2 has a significant capacity to phosphorylate recombinant α-synuclein at the serine-129 residue [67], which is the most prevalent form of α-synuclein detected in PD brains containing Lewy bodies [68]. It is suggested that the G2019S mutation in *LRRK2*, which is the most common genetic determinant of PD, has a significantly greater capacity than wild-type *LRRK2* to phosphorylate α-synuclein [67]. Given the current evidence of LRRK2 in immune response and α-synuclein phosphorylation, it is reasonable to speculate that LRRK2 may play a role in the induction of α-synuclein by mediating immune response, leading to neurodegeneration. *LRRK2* N2081D variant (rs33995883), identified to be associated with an increased risk for CD, is located in the same kinase domain as G2019S, and thus, the N2081D variant is associated with increased kinase activity like the G2019S mutation [69]. However, N551K and R1398H, the protective variants, have no effects on kinase activity. To further investigate the effect of CD- and PD-associated *LRRK2* mutations on kinase activity, Ken Y. Hui et al. quantified phosphorylation of a newly identified LRRK2 substrate, Ras-related protein 10 (Rab10), and showed that both kinase domain disease-associated mutations (G2019S and N2081D), increased the phosphorylation of Rab10 [69] which stops EHBP1L1-mediated recycling and inhibits PI3K-Akt immunological responses in phagocytic cells [70]. It has been reviewed that the role of LRRK2 regulating immune cell function is dependent on LRRK2 kinase activity [71].

**Table 2:** Genetic overlap between IBD and PD.

Genes	SNPs	Expressed position	Possible functional linkages
*LRRK2*	IBD: rs17467164, N2081D, N551K PD: G2019S, R1398H	Neurons; Glial cells; Immune cells	Increased LRRK2 activity may increase susceptibility to gut inflammation and then induce systemic inflammation, finally lead to PD development; LRRK2 may induce pathogenic α-synuclein in gut by mediating immune response, leading to neurodegeneration.
*CARD15/NOD2*	IBD: R702W, G908R, 1007fs PD: NOD2-2857A>G	Macrophages; Mucosa epithelial cells	The variants of *NOD2* are associated with aberrant host defense and decreased epithelial lymphocytes which may contribute to systemic inflammation, leading to the development of PD.
*TLR2*	IBD: R753Q, a GTn microsatellite repeat polymorphism in intron 2 PD: rs3804099, rs3804100	Intestinal epithelial cells Immune cells	The dysfunction of TLR2 is highly associated with intestinal dysbiosis which has been found to be related to development of both IBD and PD
*TLR4*	IBD: D299G, T399I PD: rs1927914	Intestinal epithelial cells Immune cells Endothelial cells Microglia cells	TLR4-mediated intestinal inflammation associated with brain inflammation can lead to neurodegeneration in PD.
*TLR9*	IBD: -1237C/T, 2848A/G, -1486CC, 1174GG, 2848AA PD: rs352140T	B cells Plasmacytoid dendritic cells Monocytes/macrophages	The variants of *TLR9* associated with inflammation may contribute to systemic inflammation which may increase risk of PD.
*Nurr1*	IBD: NA PD: rs35479735, 7048-7049insG, IVS7+33C>T, -291delT, -245T>G	Neurons Immune cells	The deletion of *Nurr1* causes exacerbation of gut inflammation and the loss of dopaminergic neurons protection.
*NLRP3*	IBD:rs10754558 rs4353135, rs4266924, rs6672995, rs10733113, rs107635144, rs55646866 PD:rs7525979	Immune cells Intestinal epithelial cells Neurons Microglia cells	The NLRP3 inflammasome can lead to disrupted brain homeostasis and brain inflammation by modulating inflammatory pathways, activating microglia and facilitating transmission of aggregated α-synuclein. The variants of NLRP3 associated with activation of astrocytes may contribute to motor abnormalities in PD.
*GBA*	IBD: rs3180018 PD:N188S, P201H, R257Q, S271G, L444P	Lysosome	*GBA* mutations associated with intestinal permeability maintenance may cause intestinal inflammation and subsequent systemic inflammation which may increase risk of PD. Together with dopaminergic cells death and microglial activation, *GBA* mutations may lead to neurodegeneration and PD.
*GPR65*	IBD: rs8005161, I231L PD: rs8005172	Lymphoid organs Peripheral blood leukocytes	The role of GPR65 in lysosomal function and pathogen defense may link IBD and PD.
*SLC39A8*	IBD: A391T PD:A391T	Plasma membrane Intracellular organelles	*SLC39A8* mutation impairs intestinal barrier, blood-brain barrier integrity and aggravates inflammatory signals, thus may contribute in gut-to-centrally mediated inflammation, leading to PD development.

SNP: single nucleotide polymorphism; IBD, inflammatory bowel disease; PD, Parkinson’s disease; *LRRK2, Leucine-rich repeat kinase 2*; *TLR, Toll-like receptor*; *Nurr1, Nuclear receptor-related factor 1*; *NLRP3, NLR Family Pyrin Domain Containing 3*; *GBA, β-glucocerebrosidase*; *GPR65, G-protein coupled receptor 65*; *SLC39A8, Solute Carrier Family 39 Member 8*

LRRK2 plays a role in inflammation regulation and pathogen defense. LRRK2 pathway is significantly enriched in response to *Mycobacterium tuberculosis* infection [72] and promotes the activation of NLRC4 inflammasome during *Salmonella* Typhimurium infection [73]. LRRK2 is involved in LPS-induced activation of TLR signaling pathway in human lymphoblasts [74]. LRRK2 is increased in patients with CD and the increase is possibly induced by interferon-γ (IFN-γ). A recent study has shown an elevated pro-inflammatory response of CD14+ monocytes from CD patients with the *LRRK2* M2397 to IFN-γ [75]. Vice versa, transgenic mice overexpressing *LRRK2* have been found exhibited more severe dextran sodium sulfate (DSS)-induced colitis than control animals [76]. Mice overexpressing pathogenic *LRRK2* mutations exhibit long-term LPS-induced nigral neuronal loss without infiltrating peripheral immune cells in the parenchyma [77]. Furthermore, neuroinflammation is not directly mediated through resident microglia, suggesting that *LRRK2* mutation mediates systemic and central immune responses which may lead to neurodegeneration.

It has yet to be established the exact role of LRRK2 in peripheral-to-centrally mediated immune signaling.

### CARD15/NOD2

NOD2, encoded by *CARD15*, is an intracellular bacterial sensor. Thus, NOD2 recognizes bacterial components, mediates the activation of nuclear factor kappa-B (NF-κB), induces or enhances apoptosis, and plays an important role in host defense and inflammation [78]. CARD15 is less expressed in the normal colon and has an excessive production in macrophages and mucosa epithelial cells of the CD colon [79].

Three polymorphisms of *CARD15/NOD2* (R702W, G908R, and 1007fs) were identified to be independently associated with the susceptibility to CD [80]. A higher frequency of total *CARD15/NOD2* gene variants has been found in PD patients than in controls [81], implicating the *CARD15/NOD2* gene as a risk factor of both CD and PD. A recent study conducted in Chinese Han population newly identified the association of *NOD2*-2857A>G polymorphism with PD susceptibility [82]. However, a genetic study performed in German case-control samples showed no association between *NOD2* variants and PD [83].

On the basis of the identification of CARD15/*NOD2* risk polymorphisms in CD, several research studies have shown interest in delineating the role of NOD2 in the intestinal mucosa. NOD2 signaling in intestinal DCs and macrophages has been shown to maintain intraepithelial lymphocytes, and the loss of NOD2 leads to a decrease in intestinal epithelial lymphocytes, which in turn predisposes mice to non- dextran sulfate sodium-induced colitis [84].

NOD2 has been found to be associated with the 6-hydroxydopamine-induced dopaminergic degeneration through oxidative stress, indicating that NOD2 promotes dopaminergic degeneration regulated by NADPH oxidase 2; thus, NOD2 can be considered as a novel innate immune signaling molecule of PD inflammatory response [85].

### TLR

TLRs are members of pattern recognition receptors expressed throughout the gastrointestinal tract in intestinal epithelial cells, myofibroblasts, enteroendocrine cells, and in various immune cells. TLRs maintain the integrity of the epithelial barrier, affect intestinal homeostasis and play an important role in immune and inflammatory responses [86, 87]. Among various TLRs, some variants of the *TLR2*, *TLR4, TLR9* genes are considered to be risk factors of IBD and PD [88, 89].

The *TLR2* gene is expressed in intestinal epithelial cells and immune cells [90]. The *TLR2* variant R753Q is related to UC and leads to the deficiency of TFF3 synthesis impairing wound healing [91]. In *TLR2* gene, a GT_n_ microsatellite repeat polymorphism in intron 2 can impact innate immune response[92]. However, a study has shown that these two polymorphisms do not have an effect on the susceptibility of IBD [93]. Two *TLR2* variants (rs3804099 and rs3804100) are associated with sporadic PD in Han Chinese population [94]. TLR2 plays an important role in intestinal homeostasis by recognizing various bacterial lipoproteins [95]. And TLR2 stimulation effectively maintains tight junction (TJ) -associated barrier and positively associates with mucin secretion [96, 97]. *TLR2 variants* may affect the immune response to bacterial stimulus, lead to intestinal inflammation and break intestinal barrier.

The *TLR4* gene is located on chromosome 9q32-q33 and is expressed in macrophages, DCs, endothelial cells, intestinal epithelium cells, and microglia in the CNS. Variants of the *TLR4* (D299G and T399I) gene have been found to be associated with the risk of IBD [88] and particular CD [98]. It has also been demonstrated that *TLR4* gene rs1927914 polymorphisms might contribute to the risk of PD in Chinese Han population [89]. LPS is a primary ligand for TLR4, and it has been shown that TLR4 is elevated in the brain of PD patients [66]. Moreover, TLR4-mediated intestinal inflammation plays an important role in brain inflammation, leading to neurodegeneration in PD [99].

TLR9 is mainly expressed in B cells, plasmacytoid DCs and monocytes/macrophages [93]. It has been shown that *TLR9* variants (-1237C/T and 2848A/G) are related to CD in a German population and *TLR9* variants (-1486CC, 1174GG and 2848AA) increase the susceptibility of UC [81, 82]. It has been indicated that the *TLR9* variant rs352140T is associated with PD [83]. Microbial DNA can stimulate immune cells as the main ligand of NLR9 and *TLR9* variants may promote the inflammation, for example, -1237C/T has been shown to have a potential binding site for NF-κB [94].

### Nurr1

The orphan nuclear receptor Nurr1 (also known as NR4A2) is an essential transcription factor affecting the generation and maintenance of dopaminergic neurons in the brain. Decrease in Nurr1 function either due to diminished expression or rare mutation is associated with PD. The protective role of Nurr1 against α-synuclein toxicity in blocking the nuclear translocation of NF-κB was observed in previous studies [100].

*Nurr1* rs35479735 polymorphism has been identified to be associated with higher risk of PD, and the results of the sequencing analysis suggest that *Nurr1* is a susceptibility gene for PD [101]. Some other variants of *Nurr1* (7048-7049insG, IVS7+33C>T, -291delT and -245T>G) are also associated with PD. Although the variant of *Nurr1* in IBD has not been identified yet, Nurr1 emerges as an important nuclear factor linking gastrointestinal inflammation and cancer [102]. It has been reported that Nurr1 can maintain T-cell homeostasis by regulating induction, maintenance, and suppressor functions of regulatory T cells (Tregs) [103]. The deletion of Nurr1 in T cells attenuates induction of Tregs and causes aberrant induction of T helper lymphocytes 1 (Th1), leading to the exacerbation of colitis [103]. Activation of Nurr1 ameliorates IBD by inducing Treg cell differentiation, suggesting Nurr1 as a promising target for treating inflammatory autoimmune diseases [104].

### NLRP3

NLRP3 is mainly expressed in immune cells [105]. The NLRP3 inflammasome has been found to be associated with inflammation- and immune-related disorders in both IBD and PD [106, 107]. The NLRP3 expression in macrophages, neutrophils, monocytes, DCs, and intestinal epithelial cells can affect IBD progression [106]. The *NLRP3* single nucleotide polymorphism (SNP) genotype of rs10754558 is significantly associated with UC [108] while SNPs rs4353135, rs4266924, rs6672995, rs10733113, rs107635144, rs55646866 are related to CD susceptibility [109]. NLRP3 inflammasome is related to the initiation and maintenance of inflammation, the variants of *NLRP3* may cause the dysfunction of the NLRP3 inflammasome, subsequently leading to IBD [106].

Inflammasome is also observed in the CNS, in response to acute infection or cell loss that occurs during neurodegeneration [110]. Moreover, α-synuclein can activate the NLRP3 inflammasome in human microglia [111]. It has been identified that the L351P *NLRP3* mutation contributes to motor abnormalities in animals by activating astrocytes [112]. However, *NLRP3* rs7525979 has been reported as an SNP associated with a significantly reduced risk of developing PD and this synonymous SNP alters the efficiency of *NLRP3* translation, thereby affecting NLRP3 protein stability, ubiquitination state, and solubility [113]. It has been reported that NLRP3 signaling is involved in the modulation of inflammatory pathways by enteric bacteria which leads to disrupted brain homeostasis [114]. And the NLRP3 inflammasome may facilitate exosome transmission of aggregated α-synuclein by promoting the generation of microvesicle by microglial cells [115].

### β-glucocerebrosidase (GBA)

GBA is a lysosomal hydrolase, encoded by the *GBA* gene that is important in α-synuclein degradation [116]. Approximately 5% - 10% of PD patients have mutant GBA alleles [117], making *GBA* mutations (including N188S, P201H, R257Q, S271G, and L444P) the commonest genetic risk factor for PD.

PD patients with *GBA* mutations have an earlier disease onset and a higher risk of dementia [118], and latest study has revealed that GBA activity is negatively correlated with level of α-synuclein [119]. PD patients have significantly higher α-synuclein levels with lower GBA activity in peripheral blood mononuclear cells [120]. The presence of GBA mutations in dopaminergic cells leads to endoplasmic reticulum stress and to their death, contributing to the development of PD [121]. Some new findings partially revealed the role of GBA in PD. A very recent study has found increased brain microglial activation in *GBA* mutation carriers without PD [122]. Jewett KA *et al.* have used drosophila model of GBA deficiency that manifests neurodegeneration and accelerated protein aggregation. They have found that GBA reduces the spread of protein aggregation by regulating proteins trafficked by extracellular vesicles [123].

*GBA* mutation has also been identified associated with IBD. It has been shown that by inhibiting GBA, glucosylceramide levels in colon organoids increase, leading to reduced colon permeability and decreased bursting [124], suggesting the possible role of GBA in maintaining intestinal permeability.

### G-protein coupled receptor 65 (GPR65, also known as TDAG8)

GPR65 is a proton-sensing G protein-coupled receptor encoded by IBD susceptibility gene [125], playing a role in maintaining lysosomal pH and lysosomal function, preserving autophagy and pathogen defense. GPR65 is highly expressed in lymphoid organs and peripheral blood leukocytes and activated by extracellular protons.

*GPR65* rs8005161 polymorphism has been found highly associated with UC and it plays an important role in pH-associated activation in intestinal inflammation [126]. IBD-associated missense variant *GPR65* I231L which expressed in epithelium cells of IBD patients displays aberrant lysosomal pH resulting in lysosomal dysfunction and impaired bacterial restriction [127]. GPR65 deficiency in DSS-induced colitis mice model triggers colonic macrophage and neutrophil infiltration and increased expression of pro-inflammatory mediators [128]. The lack of *GPR65* in epithelial cells and macrophages result in impaired clearance of bacteria and accumulation of aberrant lysosomes [127]. Though the functional role of *GPR65* variants in PD remains to be identified, abnormal regulation of autophagy is responsible for both PD and CD [129]. Further research of GPR65 will be important as it might reveal common pathogenesis of PD and IBD.

**Figure 2.**
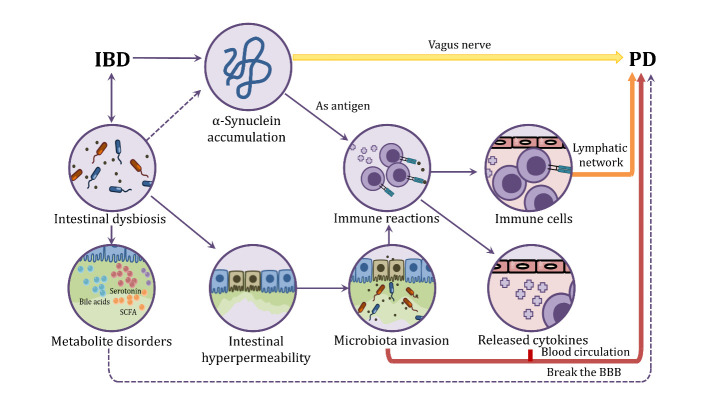
Potential linkage relationship between IBD and PD. BBB, blood-brain barrier; SCFA, short chain fatty acid.

### SLC39A8

*SLC39A8* encodes ZIP8, which is a membrane transporter responsible for manganese uptake into the cell. SLC39A8 predominantly localizes within the plasma membrane and also expresses in intracellular organelles including mitochondria [130]. A SLC39A8-dependent shift in the gut microbiome has been observed in multiple complex diseases [131].

SLC39A8 functional deletion mutations are shown in human patients with neurological and skeletal abnormalities [132, 133]. The *SLC39A8* A391T missense variant has been identified associated with CD and PD [131, 134]. Toru Nakata *et al.* identified the function of SLC39A8 in Mn homeostasis in the intestine and revealed the mechanism that A391T missence variant impairs glycocalyx and mucus barrier integrity [135]. Manganese toxicity through environmental exposure results in tremor and bradykinesia. Decreased ZIP8 results in disturbed blood-brain barrier (BBB) integrity, increased inflammatory mediator and NF-κB activation, indicating the loss of ZIP8 function may aggravate immune dysfunction [136]. Moreover, A391T allele is also in correlation with cardiovascular diseases, liver inflammation and fibrosis [137].

### Inflammation in both IBD and PD

It is generally believed that the immune system plays an active role in PD etiology and is has been hypothesized that the neuroinflammation observed in PD patients might not only be the consequence of neurodegeneration [138]. In a recent study, it has been observed that patients with PD have an increased expression of TLR4, accompanied with intestinal barrier disruption and higher pro-inflammatory factors [99]. Similar inflammatory manifestations of IBD and PD and the possible pathological process of inflammation transferring from intestinal tract to brain may reveal the pathological mechanism of PD (Fig.2).

Gut inflammation in IBD is driven mainly by the inflammatory effector CD4+ T-cell subsets, namely Th1 and T helper lymphocytes 17 (Th17) [139]. In addition, Tregs, a suppressive subset of lymphocytes which can suppress inflammation induced by Th1 and Th17, seem to play a crucial role in maintaining intestinal homeostasis [140]. Similar to the case of IBD, current evidence indicates that in patients with PD, CD4+ T cells infiltrate into the brain and the populations of peripheral blood Th1 and Th17 cells are significantly increased [141]. Moreover, Tregs could also attenuate neuroinflammation and protect neurons in PD mouse model, and a decrease in the levels of Tregs in PD patients was also found. All these findings suggest that a deficient suppression of the proinflammatory response may contribute to the pathophysiology of PD [142].

**Figure 3.**
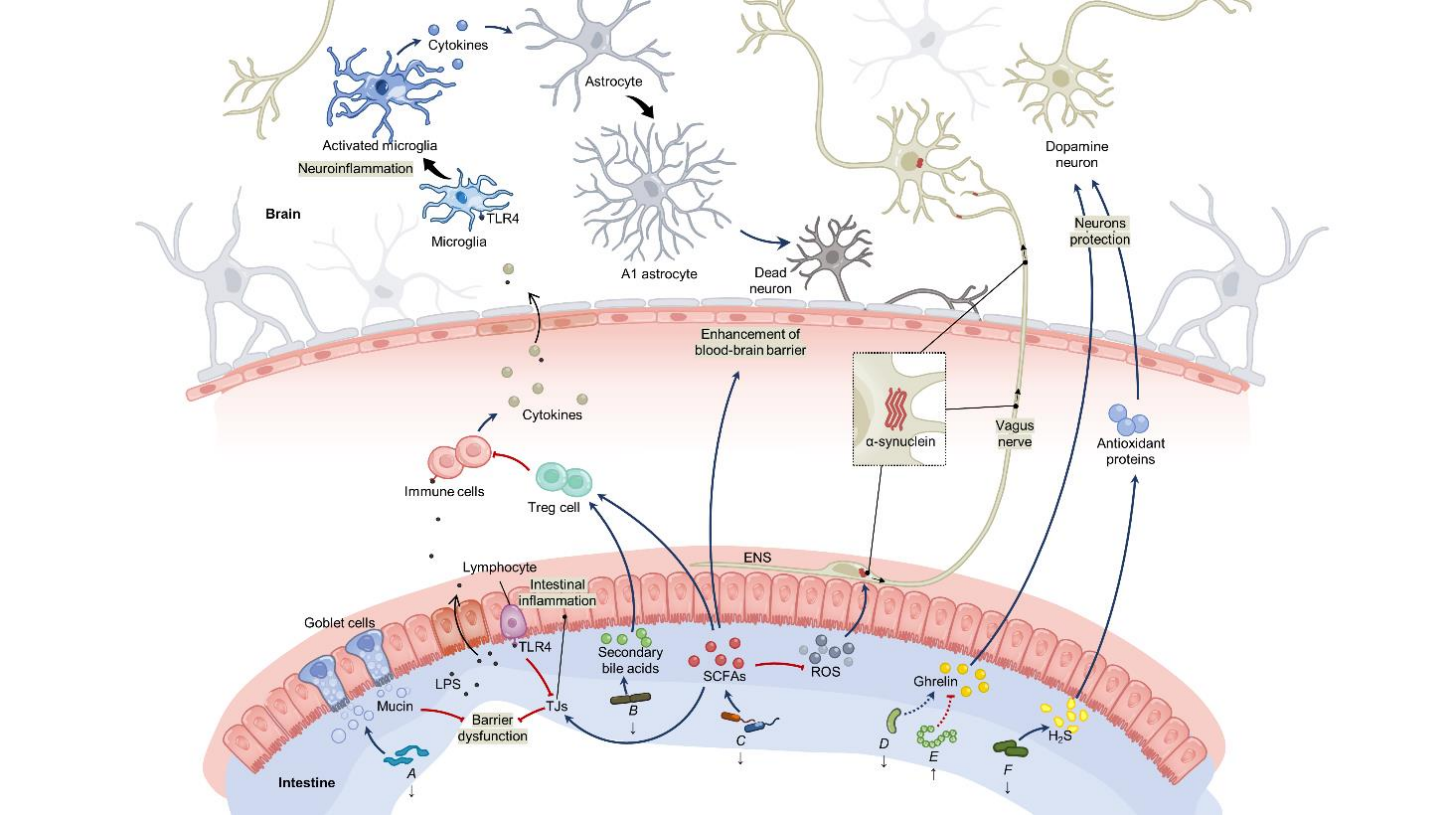
Intestinal dysbiosis in IBD contribute to PD. A refers to Revotellaceae and Ruminoccous; B refers to *Roseburia*; C, refers to Lachnospiraceae, *Roseburia*, *Faecalibacterium*, *Ruminococcus* and *Blautia*. D, E and F respectively refers to Prevotella, *Lactobacillus* and Prevotellaceae. ENS, enteric nervous system; LPS, lipopolysaccharide; TLR4, Toll-like receptor 4; TJs, tight junctions; ROS, reactive oxygen species; H2S, hydrogen sulfide; SCFAs, short chain fatty acids.

Systemic inflammation and BBB dysfunction have also been commonly reported in PD patients [143, 144]. It has been suggested that the increased intestinal permeability and systemic inflammation are sufficient to lead to disruption of BBB, brain inflammation, and ultimately alteration in brain dopaminergic function [145]. A study showed that acute DSS-induced colitis enhanced infiltration of the hippocampus with periphery immune cells, along with increased pro-inflammatory cytokines and inflammatory M1-like microglia [146]. A recent gene analysis study has provided insights into the involvement of systemic inflammation after colon injury, which is related to the degeneration process of PD [147]. Scientists combined a DSS-induced UC experimental mice model with an acute 1-methyl-4-phenyl-1,2,3,6-tetradropyridine (MPTP) intoxication. Dysfunction processes related to the blood in the striatum of UC experimental mice model and oxidative stress processes are more prominent at the MPTP intoxicated mice. Differentially expressed genes within the MPTP+DSS show functional enrichment in inflammation and programmed cell death and both toxins have a significant synergistic negative effect. Similarly, another study using MPTP+DSS mice showed significant decrease of dopaminergic neurons and dopaminergic fibers, suggesting that intestinal inflammation is able to exacerbate death of dopaminergic neurons through inducing system inflammation [148].

### Increased intestinal permeability in both IBD and PD

IBD patients have shown an increased paracellular permeability together with a downregulated TJ expression [149]. And the reduced expression levels of both occludin and ZO-1 have been reported in colon biopsies of PD patients [99, 150].

Several studies have shown a significantly elevated urinary sucralose level but normal levels of mannitol and lactulose [99], suggesting that it is the colon of PD patients that has hyperpermeability. Two fecal markers of increased intestinal permeability (alpha-1-antitrypsin and zonulin) which are increased in IBD patients have been reported significantly elevated in PD patients compared to age-matched controls [151]. Those markers are not disease-specific, but corroborate the hypothesis of an intestinal permeability as contributing factor in PD.

Increased intestinal permeability leads to greater exposure to lumen antigens and bacteria [152], which in turn triggers the immune reactions and intestinal inflammation which initiating excessive α-synuclein expression in the colon or propelling its misfolding [153].

### Intestinal dysbiosis in IBD and PD

The human intestinal microbiota is mainly composed of bacteria, fungi, and viruses, which is a dynamic and diverse community. Bacterial diversity is reduced in both IBD and PD [154, 155]. Oral antibiotics can significantly decrease the risk of PD [156]. Similar alterations of some bacterial groups between IBD and PD suggest the potential relationship between IBD and PD (Fig.3).

Compared with health control, IBD patients have a higher relative abundance of *Lactobacillus*, Entero-bacteriaceae and *Proteus* which also increase in PD patients [155, 157]. Moreover, both IBD and PD patients have a lower abundance of *Coprococcus*, *Faecalibacterium Roseburia*, Prevotellaceae, *Faecali-bacterium prausnitzii*, Lachnospiraceae and *Ruminococcus* [155, 158]. Meta-analysis of the association between the gut microbiota and PD has shown significantly lower abundance levels of *Prevotellaceae*, *Faecalibacterium*, and *Lachnospiraceae* in patients with PD [159]. Some of these microbiota changes in PD are related to motor and non-motor symptoms. The increased level of Enterobacteriaceae is associated with gait difficulty and postural instability, while decreasing level of *Lachnospiraceae* correlated with motor impairment [160].To examine microbiome alteration prior to the induction of a PD murine model, Zach Dwyer *et al.* pre-treated mice with probiotic, or inflammatory promoter DSS [161]. The DSS treatment caused marked changes in the gut microbiome and also increased the impact of LPS plus paraquat upon microglial morphology, along with circulating inflammatory markers.

Intestinal dysbiosis may be one of the potential mechanisms linking IBD and PD (Fig.3). Firstly, the expression of intestinal α-synuclein may be affected by the reduction of bacteria producing short chain fatty acids (SCFAs) like Lachnospiraceae, *Roseburia*, *Faecalibacterium*, *Ruminococcus* and *Blautia* [162-164]. SCFAs are significantly decreased in the feces of PD patients [165]. SCFAs like butyrate can regulate oxidative stress in the colonic mucosa by decreasing the reactive oxygen species which promotes the accumulation of α-synuclein in the ENS [166]. A study has presented the association between PD and SNPs in genes responsible for binding of bacterial metabolites and intestinal homeostasis, demonstrating that genetic variation in the bacterial receptor may modulate risk and age-of-onset in idiopathic PD [167].

Secondly, some intestinal microbiota which exert protective effects on neurons are decreased in IBD. The decrease of Prevotellaceae in the feces samples of PD patients have been associated with a reduction in the levels of gut hormone ghrelin [168], which has been revealed to combat neurodegeneration associated with PD. Prevotellaceae secrets hydrogen sulfide (H_2_S) which has protective effect on dopaminergic neurons in rodents PD models [169]. Since H_2_S is associated with upregulation of genes encoding antioxidant proteins, including heme oxygenase-1 and glutamate-cysteine ligase, inhaled H_2_S prevents neurodegeneration and movement disorder in a mouse model of PD [170].

Thirdly, some alterations of microbiota in IBD lead to a decreased mucin and subsequent intestinal barrier dysfunction. Intestinal barrier dysfunction permits the passage of bacteria and bacterial products such as LPS. In blood vessel, LPS activates various immune cells, leading to the production of pro-inflammatory cytokines, which consequently makes their way to the brain through the BBB [171]. TLRs expressed in microglia could also be activated by LPS, leading to increased expression of proinflammatory cytokines as well as up-regulated costimulatory molecules and major histocompatibility complex class II [172]. By secreting certain cytokines, activated microglia induce A1 astrocytes which is a subtype of reactive astrocytes abundant in various neurodegenerative diseases including PD, and A1 astrocytes lose most normal astrocytic functions but gain a new neurotoxic function, rapidly killing neurons and oligodendrocytes [173].

### Discussion

Intestinal dysfunctions, including *H. pylori* infection, SIBO, IBS, diarrhea, inflammation, IETM and dysbiosis, are the main symptoms of IBD, and these dysfunctions are more or less observed in patients with PD. The relationship between intestinal inflammation and PD has caught attention of scientists. Research are increasingly focusing on intestinal inflammation as a contributor to PD and IBD is representative. There are at least four aspects including genetic overlap, alter intestinal permeability, inflammation as well as gut microbiota, which are possible to link IBD and PD. Although it is not confirmed that intestinal dysfunction may be the initial pathological mechanisms of PD, it is also expected to develop some strategies to study in both diagnosis and treatment of neurodegenerative disease from the aspect of intestine. In fact, GV-971, as a drug approved for the treatment of Alzheimer’s disease in recent years, has also shown to alleviate PD. As we know, the main mechanisms of GV-971 for treating Alzheimer’s disease are to regulate intestinal microbiota. Finally, on the basis of the gut-brain axis and the interaction between intestinal microbiota and different kinds of diseases, it may become an exciting direction to find better ways for the manipulation of gut microbiota.
